# Case report: Treatable immune-mediated severe orthostatic hypotension in SARS-CoV-2 infection

**DOI:** 10.3389/fnins.2024.1505727

**Published:** 2025-01-07

**Authors:** Kenji Theiler, Maroussia Bronchain, Eric Grouzmann, Serge Duflon, Lorenz Hirt, Renaud Du Pasquier, Gérard Waeber, Grégoire Wuerzner, Karin Diserens, Julien F. Bally

**Affiliations:** ^1^Service of Nephrology and Hypertension, Lausanne University Hospital (CHUV) and University of Lausanne, Lausanne, Switzerland; ^2^Service of Neurology, Lausanne University Hospital (CHUV) and University of Lausanne, Lausanne, Switzerland; ^3^Laboratory of Catecholamines and Peptides, Lausanne University Hospital (CHUV) and University of Lausanne, Lausanne, Switzerland; ^4^Department of Medicine, Lausanne University Hospital (CHUV) and University of Lausanne, Lausanne, Switzerland; ^5^Acute Neurorehabilitation Unit, Department of Clinical Neurosciences, Lausanne University Hospital (CHUV) and University of Lausanne, Lausanne, Switzerland

**Keywords:** autoantibodies, COVID-19, orthostatic hypotension, intravascular immunoglobulin therapy, dysautonomia, rehabilitation

## Abstract

We report a patient with autonomic dysfunction following acute SARS-CoV-2 infection, presenting progressively worsening severe orthostatic hypotension to the point where she could no longer sit or stand. The patient experienced a delay in diagnosis after an initial misdiagnosis of a functional neurological disorder. Persistent orthostatic symptoms prompted us to re-examine the diagnosis and explore other diagnostic tools, which ultimately allowed us to identify and treat severe immune-mediated orthostatic hypotension (OH). We identified autoantibodies (AAB) targeting the autonomic nervous system. Intravascular immunoglobulin therapy, along with early, specific multi-disciplinary rehabilitation, completely resolved the symptoms. Hard-to-assess patients are often penalized by suboptimal care due to the lack of a comprehensive patient history and physical examination, resulting in unnecessary and costly ancillary examinations that lead to delays in diagnosis or misdiagnoses. Furthermore, a lack of awareness of rare complications with new diseases may also hamper proper patient care. In the present case, this includes the wide range of SARS-CoV-2 infection manifestations, including immune-mediated autonomic complications.

## Introduction

Long-term complications resulting from heterogeneous manifestations after SARS-CoV-2 infection are referred to as long-haul coronavirus disease (COVID-19). The most common neurological and neuropsychiatric symptoms include fatigue, memory and concentration disorders, sleep disturbance, anxiety, and depression (Premraj et al., 2022). Cardiovascular autonomic dysfunction includes postural orthostatic tachycardia syndrome, orthostatic hypotension (OH), and neurocardiogenic syncope (Premraj et al., 2022; Jamal et al., 2022; Dani et al., 2021; Shouman et al., 2021; Bisaccia et al., 2021; Goodman et al., 2021). More recent studies have reported the presence of autonomic dysfunction as a notable early manifestation of SARS-CoV-2 infection (Scala et al., 2022a,b; Bellavia et al., 2021), even in mild cases, with a high prevalence of OH. COVID-19-positive patients exhibited more dysautonomia, particularly orthostatic hypotension, compared to COVID-19-negative controls (Scala et al., 2022a). Although techniques for measuring autonomic dysfunction have been developed (Scala et al., 2022b; Bellavia et al., 2021) (e.g., the COMPASS-31 questionnaire, Heart rate variability, Sudoscan, or pupillometry parameters), few studies have explored the physiopathology of autonomic dysfunction caused by SARS-CoV-2.

Our patient initially presented with mild SARS-CoV-2 infection and progressively worsening severe orthostatic symptoms, to the point where she could no longer sit or stand. The physical examination performed in the emergency department was limited to the supine position, and the differential diagnosis led to a diagnosis of functional neurologic disorder after ruling out other conditions, rather than being based on the observation of positive functional signs. The patient could not be examined in a standing position. A multidisciplinary workup confirmed severe OH and autonomic dysfunction. Ultimately, the patient was diagnosed with organic autoimmune-mediated orthostatic hypotension, with autoantibodies targeting the autonomic nervous system (ANS) and the renin-angiotensin-aldosterone system. She was treated appropriately and had an excellent outcome. To the best of our knowledge, this is the first report documenting the progression from diagnosis to treatment to recovery of autonomic dysfunction caused by SARS-CoV-2.

## Case description

A 43-year-old Caucasian woman with no prior medical history presented to the emergency department with the sudden, transient appearance of a black veil over the eyes and an inability to interact, without loss of consciousness. She presented no other symptoms. She had received three doses of Moderna’s SARS-CoV-2 mRNA vaccine (her last shot was three months before the symptom onset). The patient was not taking any medication that affect autonomic parameters. At rest and in a supine position, her blood pressure was 132/68 mmHg, with a heart rate of 68 beats per min. The neurological examination in the supine position did not reveal any abnormalities. However, three attempts to perform the Schellong test were unsuccessful due to severe orthostatic symptoms and signs of syncope threat (pallor and dysarthria), requiring the patient to be laid down to obtain blood pressure measurements. The laboratory results showed a normal blood count and chemistry, with no signs of inflammation, and only slightly elevated liver and pancreatic enzymes. The result of the nasopharyngeal SARS-CoV-2 PCR test was positive (2.4 ×10^8^ copies/mL). The electrocardiogram and brain MRI were both normal.

**Figure 1:**
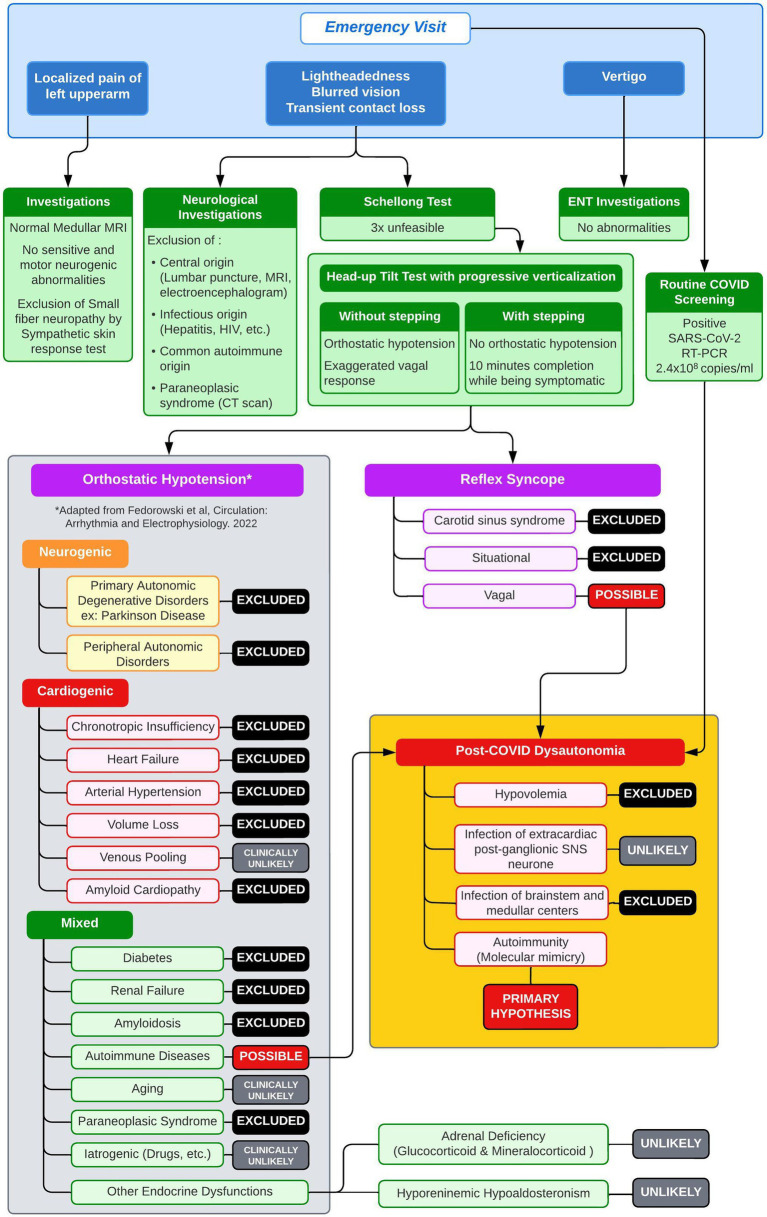
Diagnostic workup with differential diagnosis of orthostatic hypotension.

Due to her inability to walk and only occasional bouts of sitting, our patient was admitted to a nearby medical center for observation on day 4. Then, without a clear diagnosis but worsening symptoms, she was transferred to the neurology department of our tertiary care facility on day 11. The patient underwent additional tests, including autoimmune, neuro-inflammatory, and metabolic evaluations, as well as a chest–abdomen scan, lumbar puncture, electroencephalogram, electromyogram, and whole-spine magnetic resonance imaging. Neurological disorders affecting the nervous system or inner ear, as well as related infections, were ruled out (Figure 1). The routine and infectious tests of the plasma and cerebrospinal fluid were negative.

The patient’s medical history was complicated by a headache thought to be caused by a ‘migraine-like’ condition, which made it difficult for the patient to answer questions and participate in the neurological exam. She displayed signs of psychomotor slowness, cold limbs, and impaired balance due to persistent orthostatic intolerance. Attempts to conduct the Schellong test were unsuccessful, and a scheduled tilt test was canceled when a diagnosis of functional neurological disorder was established on day 15.

**Figure 2:**
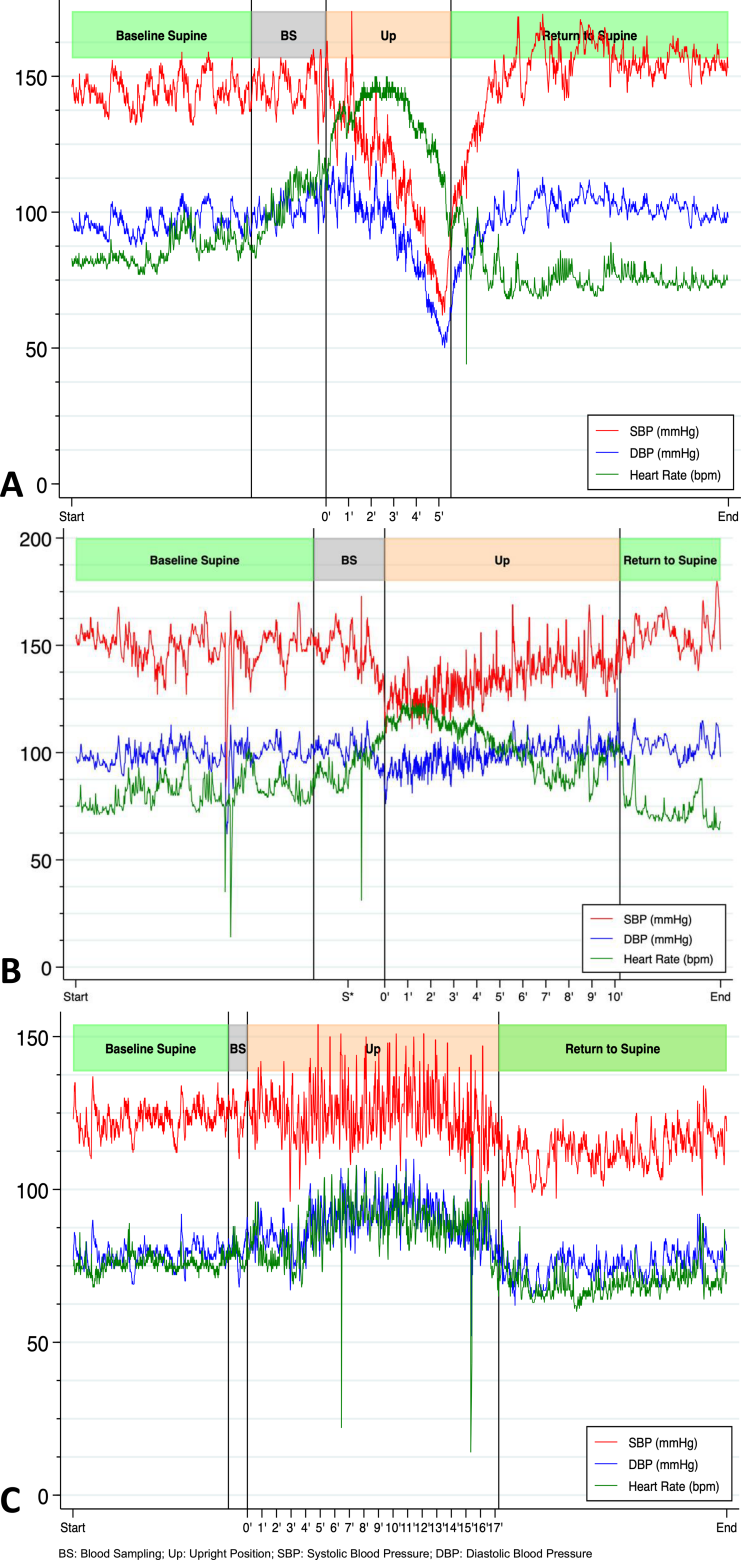
Beat-by-beat blood pressure and heart rate during first HUTT-pv performed on day 27 without stepping and before IVIG treatment **(A)**, on day 27 with stepping **(B)**, and three months after discharge without stepping **(C)** showing a completely physiological response (increase in DBP and HR, stable SBP) (Goldstein, 2021).

Our patient entered the rehabilitation program but was unable to stand, which hampered her progress. To gain a better understanding of the persistent orthostatic symptoms in the patient, who had not had a successful Schellong test since the onset of symptoms, our team conducted a head-up tilting test with progressive verticalization (HUTT-pv) on day 27 after the symptom onset, using a novel device for automated stepping training (Erigo®). The detailed method of the beat-by-beat orthostatic challenge with the HUTT-pv can be found in the supplementary material. The patient performed the test wearing compressive stockings and without stepping (Figure 2, panel A). The results showed an initial massive reactional tachycardia (from 85 beats per min (BPM) to 145 BPM), with only a slight decrease in blood pressure at 70° of verticalization during the first two min. After three min, the reactional tachycardia could no longer maintain adequate cardiac output (shown in Supplementary Figure 1), accompanied by a continual drop in blood pressure. The heart rate then dropped substantially after the fourth min, falling below 100 BPM by the fifth min. Clinical signs of syncope threat prompted us to stop the test after five min. The patient showed signs of vigilance fluctuation, dysarthria, pallor, and head drooping and complained of vertigo, suggesting decreased blood flow in the brainstem. Her blood pressure was 66/52 mmHg. During the verticalization process, the norepinephrine levels increased from an initial 1.47 nmoL/L to 3.34 nmoL/L.

The patient repeated the test 30 min later, this time with passive stepping. She was able to maintain an upright posture for 10 min without any significant decrease in diastolic blood pressure (DBP). She only experienced mild orthostatic symptoms (Figure 2, panel B).

We eliminated most causes of orthostatic hypotension (Figure 1) and hypothesized that the orthostatic hypotension was caused by an immunological disorder, triggered by the SARS-CoV-2 infection (or, less likely, by its vaccine). We initiated a 5-day course of intravenous immunoglobulin (IVIG) therapy at a dose of 0.4 g/kg/day, starting on day 28. Symptom improvement was rapid, as evidenced by a normal HUTT-pv on day 42. The patient underwent intensive rehabilitation during the same period. She was discharged and able to stand, walk, and jump without experiencing dizziness.

Before receiving IVIG, autoantibody (AAB) screening of the patient’s serum was performed, which eventually revealed the presence of eight AABs, predominantly targeting the autonomic nervous system (ANS) and the renin-angiotensin-aldosterone system (RAAS) (Table 1). This discovery provided evidence of an immune-based explanation for the symptoms and correlated with the favorable clinical outcome following IVIG treatment.

**Table 1:** Positive autoantibodies and their supposed agonist effects.

Positive autoantibodies	Units/ml	Normal value cutoff	Supposed effects of the autoantibody (if agonist)
Anti-ACE-2	18.8	<9.8 U/mL	Decrease in soluble ACE2 activity and increase in angiotensin II
Anti-MAS1	43.3	<25.0 U/mL	RAAS-specific, negative chronotropic response
Anti-Alpha-2-adrenergic-R	21.1	<15.0 U/mL	Decrease in sympathetic activity and BP
Anti-Muscarinic M1R, M2R, and M5R (partially adapted from Saternos et al. (2018)	M1: 16.1 M2: 11.3 M5: 16.4	<9.0 U/mL <9.0 U/mL <14.2 U/mL	M1: Increase in HR and contractile force, modulation of vascular tone M2: Negative chronotropic effect, vasodilation M5: Cardiovascular effects less studied
Anti-TS-HDS-IgM	9.8	<9.0 U/mL	Implicated in small fibre neuropathy and dysautonomia
Anti-PAR1	5.6	<4.2 U/mL	Role in platelet activation, endothelial smooth muscle contraction

**Figure 3:**
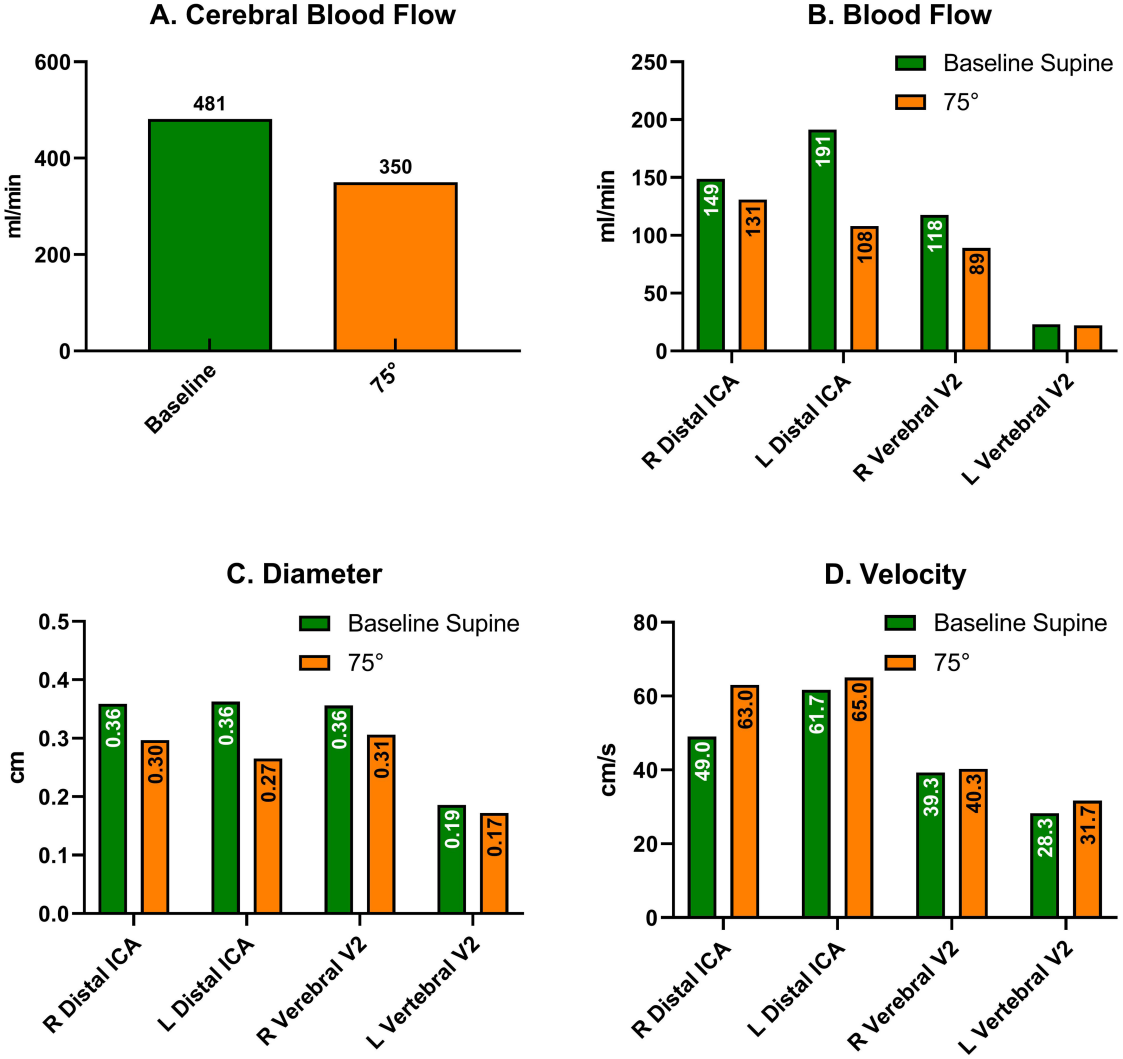
Cervical Doppler artery ultrasound in supine (green) and upright (orange) positions without stepping, three months after discharge after new orthostatic intolerance. **(A)** Cerebral Blood Flow; Blood flow **(B)**, Diameter **(C)** and Velocity **(D)** in each artery comparing supine and upright (70°) positions. ICA: Internal Carotid Artery.

Three months after discharge, the patient again reported fatigue, lack of concentration, and depressive symptoms. During a new HUTT-pv (Figure 2, panel C), lasting 17 min in a passive standing position, she experienced mild orthostatic symptoms without a significant drop in blood pressure. At the same time, a carotid artery Doppler ultrasound showed a 27% decrease in cerebral blood flow (CBF) when upright (Figure 3). No further AAB tests were performed. After receiving outpatient rehabilitation therapy in our long-term COVID-19 consultation, the patient made a full recovery and returned to work by the follow-up appointment 15 months later, with all symptoms resolved.

## Discussion

### Autonomic dysfunction associated with SARS-CoV-2 infection

SARS-CoV-2 infection is linked to a wide range of non-respiratory symptoms, from the initial phase of the infection to several months after the acute phase, commonly referred to as long-haul COVID-19. A systematic review (Scala et al., 2022b) revealed that even in non-critically ill patients, acute SARS-CoV-2 infections can cause autonomic impairment, leading to a complex imbalance between the sympathetic and parasympathetic nervous systems. Furthermore, an observational study reported a higher prevalence of OH in acute COVID-19 patients compared to a healthy control group (Scala et al., 2022a).

In the acute phase, our patient experienced severe mixed orthostatic hypotension, characterized by impairment of both the autonomic nervous system and the cardiovascular system (Fedorowski et al., 2022). The results of the HUTT-pv performed on day 27 suggested that the physiological baroreflex was preserved (as evidenced by initial tachycardia and a transient slight elevation in DBP). Verticalization triggers norepinephrine secretion in healthy volunteers and is strongly associated with diastolic blood pressure, reflecting the efferent sympathetic activation that controls vascular tone (Bahjaoui-Bouhaddi et al., 2000). Our patient behaved differently as the increased norepinephrine concentration observed during the orthostasis was eventually associated with an inappropriate decrease in diastolic blood pressure and a drop in the heart rate. These findings suggest an imbalance between the sympathetic and parasympathetic systems. Moreover, the HUTT-pv with passive stepping, which was repeated after 30 min of rest in the supine position, allowed our patient to stay verticalized for 10 min without a significant drop in DBP, experiencing only mild orthostatic symptoms. The increase in norepinephrine was lower with the passive stepping than without, indicating diminished activation of the sympathetic nervous system.

### Autoimmune causes of orthostatic hypotension and their relationship with SARS-CoV-2 infection

Orthostatic intolerance and autonomic disorders, such as OH and postural orthostatic tachycardia syndrome, are commonly reported in individuals with long-haul COVID-19 (Jamal et al., 2022; Shouman et al., 2021; Eldokla and Ali, 2022; Buoite Stella et al., 2022; Monaghan et al., 2022; Carmona-Torre et al., 2022; Eslami et al., 2023). However, research on these symptoms during acute SARS-CoV-2 infection has been limited. Regardless of SARS-CoV-2 infection, neurogenic OH and postural orthostatic tachycardia syndrome have been linked to the presence of AABs against adrenergic and muscarinic receptors, suggesting an immune origin. Goldstein (Goldstein, 2021) stated three main hypotheses for orthostatic intolerance in long-haul COVID-19: hypovolaemia, infection of extra-cardiac postganglionic sympathetic nervous system neurons by the SARS-CoV-2 virus, and autoimmunity. Common causes of cardiogenic and neurogenic OH were ruled out in our patient based on the clinical examinations, laboratory analyses, and imaging (Figure 1). Baroreflex function was preserved in the initial HUTT-pv. Although drug-induced OH is common, it was unlikely in this case, especially as there was no change in her medication following IVIG treatment. After conducting a thorough evaluation, an immunological cause was suspected, as depicted in Figure 1. The discovery of AABs targeting both the sympathetic and parasympathetic nervous systems and the RAAS confirmed that an autoimmune mechanism was in play. In addition, the rapid recovery after the IVIG treatment supported our hypothesis.

Several studies have described AABs targeting G protein-coupled receptors in the ANS and RAAS in patients with long-haul COVID-19 (Wallukat et al., 2021; Skiba and Kruse, 2021; Fedorowski et al., 2017). One study found that all 31 of its participants with long-haul COVID-19 had between two and seven different AABs against G protein-coupled receptors (Wallukat et al., 2021). Of these, 17 developed cardiovascular or neurological disorders. Our patient had eight of the 18 AABs in the panel, including those that target the ANS and RAAS, as described by Wallukat et al. (2021). However, it remains unclear whether the autoantibodies we found have functional agonist, antagonist, or modulatory effects on G protein-coupled receptor activation *in vivo* (Skiba and Kruse, 2021; Fedorowski et al., 2017). Therefore, cell-based bioassays are needed to assess the characteristics of each AAB found in our patient. To the best of our knowledge, studies associating the presence of AABs with autonomic dysfunction mainly focus on long-haul COVID-19 patients. Whether this same mechanism operates in the acute phase of the infection remains uncertain.

Our patient had autoantibodies targeting MAS1 and ACE2, potentially affecting the RAAS balance. The classic RAAS pathway increases blood pressure through angiotensin II-mediated vasoconstriction, aldosterone release, and sympathetic nervous system activation. The alternative ACE2/angiotensin-(1–7)/MAS1 axis serves as a modulator (Santos et al., 2018).

Muscarinic acetylcholine receptors are G protein-coupled receptors with five subtypes, M1R–M5R. They are widely distributed and have crucial functions in the parasympathetic nervous system. Our patient tested positive for M1R, M2R, and M5R AABs, similar to the majority of Wallukat’s 31 SARS-CoV-2 infected patients (Wallukat et al., 2021).

Viral infections can cause myalgic encephalomyelitis/chronic fatigue syndrome (ME/CFS), leading to autonomic dysregulation. Long-haul COVID-19 shares symptoms with ME/CFS (Sukocheva et al., 2022), and studies have found increased β2-adrenergic receptors and muscarinic M3R and M4R AABs in patients with CFS. Both groups experience orthostatic intolerance due to reduced CBF. Van Campen et al. found a 33 and 29% decrease in CBF in patients with long-haul COVID-19 and ME/CFS, respectively, while controls had a 4% decrease (Campen et al., 2022). Three months after discharge, our patient experienced mild orthostatic intolerance. Although the HUTT-pv was entirely normal, CBF decreased by 27% upon standing (Figure 3), again suggesting autonomic dysfunction, although mild enough not to decrease BP upon standing, and this finding was consistent with previous literature on long-haul COVID-19 and ME/CFS.

### Orthostatic hypotension rehabilitation: correlation between paraclinical results and clinical observations in the HUTT-pv on Erigo®

Inactivity leads to deconditioning, including reduced blood volume, which can occur within a few days of bed rest. Exercise increases blood volume, alleviates postural orthostatic tachycardia syndrome and OH symptoms (Raman et al., 2022; Fu et al., 2010; Johansson et al., 2021), and prevents further deconditioning (Freeman et al., 2018). Sympathetic nerve dysfunction can also contribute to orthostatic intolerance after prolonged inactivity (Wyller et al., 2008). Dietz et al. demonstrated that passive leg movement during a tilt-table test prevented benign syncope in healthy adults (Czell et al., 2004). They developed Erigo®, an automated stepping device that allows simultaneous progressive verticalization (Colombo et al., 2005). Our institution’s interdisciplinary acute neurorehabilitation unit conducted a feasibility study (Rocca et al., 2016) with Erigo®, allowing patients to safely reach a 70° upright position through passive stepping. Despite initial concerns about syncope, our patient completed a 10-min HUTT-pv with the benefit of passive stepping and experienced minimal orthostatic symptoms. Indeed, using a robotic device like Erigo® may be considered in severe OH cases, allowing for the diagnosis of OH. When coupled with blood pressure measurements correlated to the precise documentation of the degree of verticalization and the intensity and duration of training sessions involving passive stepping movements, Erigo® becomes a reproducible and quantifiable tool. It allows for evaluator-independent diagnosis and, especially, enables adequate rehabilitation despite OH.

The ability to observe the patient during the acute neurorehabilitation sessions and confirm the diagnosis using this robotic device makes this case unique as without this interdisciplinary approach in the very acute phase, these symptoms would have been considered “functional.”

## Conclusion

SARS-CoV-2 infection can trigger severe autonomic dysfunction due to autoantibodies targeting the autonomic nervous system and the renin-angiotensin-aldosterone system. Our patient, a healthy 43-year-old woman, presented with a mild SARS-CoV-2 infection and worsening orthostatic hypotension, which was initially misdiagnosed as a functional neurological disorder.

Erigo allows progressive verticalization and passive leg movement and is useful for both diagnosing and treating severe OH. Furthermore, rehabilitation with Erigo can start early, even in patients who cannot stand or walk. When combined with beat-by-beat blood pressure monitoring, this technology allows for linking clinical symptoms to quantitative data.

Upon further investigation, our team found evidence of autonomic dysfunction (severe orthostatic hypotension due to an imbalance between the sympathetic and parasympathetic nervous systems) in the initial stages of the patient’s COVID-19 infection. The patient most likely experienced immune-mediated orthostatic symptoms, as evidenced by the presence of antibodies against RAAS and ANS antigens. Her symptoms improved after 5 days of IVIG therapy. The specific roles and mechanisms of action of each autoantibody are not yet known and require further investigation, including exploring their potential overlap with other conditions such as ME/CFS, which can also lead to autonomic dysfunction.

## Data Availability

The datasets presented in this article are not readily available because of ethical and privacy restrictions. Requests to access the datasets should be directed to the corresponding author/s.

## References

[ref1] Bahjaoui-Bouhaddi M. Cappelle S. Henriet M.-T. Dumoulin G. Wolf J.-P. Regnard J. (2000). Graded vascular autonomic control versus discontinuous cardiac control during gradual upright tilt. J. Auton. Nerv. Syst. 79, 149–155. doi: 10.1016/S0165-1838(99)00068-5, PMID: 10699646

[ref2] Bellavia S. Scala I. Luigetti M. Brunetti V. Gabrielli M. Zileri Dal Verme L.. (2021). Instrumental evaluation of COVID-19 related Dysautonomia in non-critically-ill patients: an observational, cross-sectional study. J. Clin. Med. 10:5861. doi: 10.3390/jcm10245861, PMID: 34945155 PMC8703676

[ref3] Bisaccia G. Ricci F. Recce V. Serio A. Iannetti G. Chahal A. A.. (2021). Post-acute sequelae of COVID-19 and cardiovascular autonomic dysfunction: what do we know? J. Cardiovas. Dev. Dis. 8:156. doi: 10.3390/jcdd8110156, PMID: 34821709 PMC8621226

[ref5] Buoite Stella A. Furlanis G. Frezza N. A. Valentinotti R. Ajcevic M. Manganotti P. (2022). Autonomic dysfunction in post-COVID patients with and witfhout neurological symptoms: a prospective multidomain observational study. J. Neurol. 269, 587–596. doi: 10.1007/s00415-021-10735-y, PMID: 34386903 PMC8359764

[ref6] Campen C. Van M. C. Rowe P. C. Visser F. C. (2022). Orthostatic symptoms and reductions in cerebral blood flow in long-haul COVID-19 patients: similarities with Myalgic encephalomyelitis/chronic fatigue syndrome. Medicina 58:28. doi: 10.3390/medicina58010028 PMC8778312 35056336

[ref7] Carmona-Torre F. Mínguez-Olaondo A. López-Bravo A. Tijero B. Grozeva V. Walcker M.. (2022). Dysautonomia in COVID-19 patients: a narrative review on clinical course. Diagnostic and Therapeutic Strategies. Front. Neurol. 13:886609. doi: 10.3389/fneur.2022.886609, PMID: 35720084 PMC9198643

[ref8] Colombo G. Schreier R. Mayr A. Plewa H. Rupp R. (2005). Novel tilt table with integrated robotic stepping mechanism: design principles and clinical application. In: 9th international conference on rehabilitation robotics, 2005. ICORR 2005, 227–230. doi: 10.1109/ICORR.2005.1501091

[ref9] Czell D. Schreier R. Rupp R. Eberhard S. Colombo G. Dietz V. (2004). Influence of passive leg movements on blood circulation on the tilt table in healthy adults. J. Neuroeng. Rehabil. 1:4. doi: 10.1186/1743-0003-1-4, PMID: 15679913 PMC544951

[ref10] Dani M. Dirksen A. Taraborrelli P. Torocastro M. Panagopoulos D. Sutton R.. (2021). Autonomic dysfunction in ‘long COVID’: rationale, physiology and management strategies. Clin. Med. 21, e63–e67. doi: 10.7861/clinmed.2020-0896, PMID: 33243837 PMC7850225

[ref11] Eldokla A. M. Ali S. T. (2022). Autonomic function testing in long-COVID syndrome patients with orthostatic intolerance. Auton. Neurosci. 241:102997. doi: 10.1016/j.autneu.2022.102997, PMID: 35679657

[ref12] Eslami M. Mollazadeh R. Mirshafiee S. Sehat P. Alizadeh F. Emkanjoo Z.. (2023). Postural orthostatic tachycardia syndrome and orthostatic hypotension post COVID-19. Infect. Disord. Drug Targets. 23:e100622205846. doi: 10.2174/1871526522666220610143504 35692134

[ref13] Fedorowski A. Li H. Yu X. Koelsch K. A. Harris V. M. Liles C.. (2017). Antiadrenergic autoimmunity in postural tachycardia syndrome. EP Europace 19, 1211–1219. doi: 10.1093/europace/euw154, PMID: 27702852 PMC5834103

[ref14] Fedorowski A. Ricci F. Hamrefors V. Sandau K. E. Hwan Chung T. Muldowney J. A. S.. (2022). Orthostatic hypotension: Management of a Complex, but common, Medical Problem. Circulation 15:e010573. doi: 10.1161/CIRCEP.121.010573, PMID: 35212554 PMC9049902

[ref15] Freeman R. Abuzinadah A. R. Gibbons C. Jones P. Miglis M. G. Sinn D. I. (2018). Orthostatic hypotension. J. Am. Coll. Cardiol. 72, 1294–1309. doi: 10.1016/j.jacc.2018.05.079, PMID: 30190008

[ref16] Fu Q. VanGundy T. B. Galbreath M. M. Shibata S. Jain M. Hastings J.. (2010). Cardiac origins of the postural orthostatic tachycardia syndrome. J. Am. Coll. Cardiol. 55, 2858–2868. doi: 10.1016/j.jacc.2010.02.043, PMID: 20579544 PMC2914315

[ref17] Goldstein D. S. (2021). The possible association between COVID-19 and postural tachycardia syndrome. Heart Rhythm. 18, 508–509. doi: 10.1016/j.hrthm.2020.12.007, PMID: 33316414 PMC7729277

[ref18] Goodman B. P. Khoury J. A. Blair J. E. Grill M. F. (2021). COVID-19 Dysautonomia. Front. Neurol. 12:624968. doi: 10.3389/fneur.2021.624968, PMID: 33927679 PMC8076737

[ref19] Jamal S. M. Landers D. B. Hollenberg S. M. Turi Z. G. Glotzer T. V. Tancredi J.. (2022). Prospective evaluation of autonomic dysfunction in post-acute sequela of COVID-19. J. Am. Coll. Cardiol. 79, 2325–2330. doi: 10.1016/j.jacc.2022.03.357, PMID: 35381331 PMC8976261

[ref20] Johansson M. Ståhlberg M. Runold M. Nygren-Bonnier M. Nilsson J. Olshansky B.. (2021). Long-haul post–COVID-19 symptoms presenting as a variant of postural orthostatic tachycardia syndrome: the Swedish experience. JACC 3, 573–580. doi: 10.1016/j.jaccas.2021.01.009, PMID: 33723532 PMC7946344

[ref21] Monaghan A. Jennings G. Xue F. Byrne L. Duggan E. Romero-Ortuno R. (2022). Orthostatic intolerance in adults reporting long COVID symptoms was not associated with postural orthostatic tachycardia syndrome. Front. Physiol. 13:833650. doi: 10.3389/fphys.2022.833650, PMID: 35309052 PMC8931464

[ref22] Premraj L. Kannapadi N. V. Briggs J. Seal S. M. Battaglini D. Fanning J.. (2022). Mid and long-term neurological and neuropsychiatric manifestations of post-COVID-19 syndrome: a meta-analysis. J. Neurol. Sci. 434:120162. doi: 10.1016/j.jns.2022.120162, PMID: 35121209 PMC8798975

[ref23] Raman B. Bluemke D. A. Lüscher T. F. Neubauer S. (2022). Long COVID: post-acute sequelae of COVID-19 with a cardiovascular focus. Eur. Heart J. 43, 1157–1172. doi: 10.1093/eurheartj/ehac031, PMID: 35176758 PMC8903393

[ref24] Rocca A. Pignat J.-M. Berney L. Jöhr J. van de Ville D. Daniel R. T.. (2016). Sympathetic activity and early mobilization in patients in intensive and intermediate care with severe brain injuries: a preliminary prospective randomized study. BMC Neurol. 16:169. doi: 10.1186/s12883-016-0684-2, PMID: 27619015 PMC5020460

[ref25] Santos R. A. S. Sampaio W. O. Alzamora A. C. Motta-Santos D. Alenina N. Bader M.. (2018). The ACE2/angiotensin-(1–7)/MAS Axis of the renin-angiotensin system: focus on angiotensin-(1–7). Physiol. Rev. 98, 505–553. doi: 10.1152/physrev.00023.2016, PMID: 29351514 PMC7203574

[ref26] Saternos H. C. Almarghalani D. A. Gibson H. M. Meqdad M. A. Antypas R. B. Lingireddy A.. (2018). Distribution and function of the muscarinic receptor subtypes in the cardiovascular system. Physiol. Genomics 50, 1–9. doi: 10.1152/physiolgenomics.00062.2017, PMID: 29093194

[ref27] Scala I. Bellavia S. Luigetti M. Brunetti V. Broccolini A. Gabrielli M.. (2022a). Autonomic dysfunction in non-critically ill COVID-19 patients during the acute phase of disease: an observational, cross-sectional study. Neurol. Sci. 43, 4635–4643. doi: 10.1007/s10072-022-06136-2, PMID: 35608736 PMC9127042

[ref28] Scala I. Rizzo P. A. Bellavia S. Brunetti V. Colò F. Broccolini A.. (2022b). Autonomic dysfunction during acute SARS-CoV-2 infection: a systematic review. J. Clin. Med. 11:3883. doi: 10.3390/jcm11133883, PMID: 35807167 PMC9267913

[ref29] Shouman K. Vanichkachorn G. Cheshire W. P. Suarez M. D. Shelly S. Lamotte G. J.. (2021). Autonomic dysfunction following COVID-19 infection: an early experience. Clin. Auton. Res. 31, 385–394. doi: 10.1007/s10286-021-00803-8, PMID: 33860871 PMC8050227

[ref30] Skiba M. A. Kruse A. C. (2021). Autoantibodies as endogenous modulators of GPCR signaling. Trends Pharmacol. Sci. 42, 135–150. doi: 10.1016/j.tips.2020.11.013, PMID: 33358695 PMC7880908

[ref31] Sukocheva O. A. Maksoud R. Beeraka N. M. Madhunapantula S. R. V. Sinelnikov M. Nikolenko V. N.. (2022). Analysis of post COVID-19 condition and its overlap with myalgic encephalomyelitis/chronic fatigue syndrome. J. Adv. Res. 40, 179–196. doi: 10.1016/j.jare.2021.11.013, PMID: 36100326 PMC8619886

[ref32] Wallukat G. Hohberger B. Wenzel K. Fürst J. Schulze-Rothe S. Wallukat A.. (2021). Functional autoantibodies against G-protein coupled receptors in patients with persistent long-COVID-19 symptoms. J. Transl. Autoimmunity 4:100100. doi: 10.1016/j.jtauto.2021.100100, PMID: 33880442 PMC8049853

[ref33] Wyller V. B. Saul J. P. Walløe L. Thaulow E. (2008). Sympathetic cardiovascular control during orthostatic stress and isometric exercise in adolescent chronic fatigue syndrome. Eur. J. Appl. Physiol. 102, 623–632. doi: 10.1007/s00421-007-0634-1, PMID: 18066580

