# Supplementary material for: Case report: Treatable immune-mediated severe orthostatic hypotension in SARS-CoV-2 infection
Source: Front Neurosci. 2025 Jan 7;18:1505727. doi: 10.3389/fnins.2024.1505727 (PMC11746903; doi:10.3389/fnins.2024.1505727)
Supplement: Supplementary file 1 [file Data_Sheet_1.docx]

Supplemental Material

[1. Methods 2](#_Toc152346047)

[1.1. Continuous measurement of haemodynamic parameters 2](#_Toc152346048)

[1.2. Head-up-tilt-test with progressive verticalization (HUTT-pv) on Erigo® 2](#_Toc152346049)

[1.3. Measurement of Catecholamines, PRA and Aldosterone 2](#_Toc152346050)

[1.4. Auto-antibody screening 2](#_Toc152346051)

[2. Results 2](#_Toc152346052)

[2.1. Day 27 HUTT-pv without stepping and before IVIG (Figure 2, panel A) 2](#_Toc152346053)

[2.2. Day 42 HUTT-pv without stepping and after IVIG 3](#_Toc152346054)

[2.3. Results of HUTT-pv with stepping at Day 27 (supplemental Figure 2) and Day 42 3](#_Toc152346055)

[2.4. Results of HUTT-pv without stepping three months after discharge (Figure 2, Panel B) 3](#_Toc152346056)

[2.5. Laboratory Analysis 3](#_Toc152346057)

[2.6. Extensive assessment 4](#_Toc152346058)

[2.6.1. Thoraco-abdominal CT-scan 4](#_Toc152346059)

[2.6.2. Brain MRI 4](#_Toc152346060)

[2.6.3. Total medullar MRI 4](#_Toc152346061)

[2.6.4. Lumbar punctures 4](#_Toc152346062)

[2.6.5. Blood analysis 5](#_Toc152346063)

[2.6.6. Electroencephalogram 5](#_Toc152346064)

[2.6.7. Electroneuromyography (ENMG) 5](#_Toc152346065)

[2.7. Supplemental figures 6](#_Toc152346066)

[2.7.1. Supplemental figure 1: Day-27 Beat-by-beat measurement of heart rate, blood pressure and cardiac output (Same as figure 2, panel A, but increased zooming in and addition of relative cardiac output to baseline) 6](#_Toc152346067)

[2.7.2. Supplemental figure 2: Beat-by-beat measurement of heart rate and blood pressure on Day-27-HUTT-pv with stepping, showing no orthostatic hypotension. 6](#_Toc152346068)

[3. Citations 7](#_Toc152346069)

# Methods

## Continuous measurement of haemodynamic parameters

We obtained beat-by-beat measurement of haemodynamic parameters by Finapres® NOVA (Finapres Medical Systems, Enschede, The Netherlands) with volume clamp finger plethysmography calibrated with an automated arm cuff oscillometric blood pressure measurement.

## Head-up-tilt-test with progressive verticalization (HUTT-pv) on Erigo®

The HUTT-pv with Erigo® was performed with beat-by-beat blood pressureandheart rate monitoring associated with blood sampling. The patient was attached to the verticalization-robot Erigo® (Hocoma AG, Switzerland) in supine position. Erigo® progressively verticalized the patient to a maximum of 70°, first without passive stepping (figure 1), then while continuously and passively stepping the patient. Baseline supine measurement was realized for 10 minutes followed by progressive verticalization for 10 minutes, or shorter in case of syncope. Recording was continued for another 15 minutes after return to supine position.

## Measurement of Catecholamines, PRA and Aldosterone

Sympathetic activity was followed by plasma catecholamines (epinephrine and norepinephrine) levels, measured by highly sensitive ultra-performance liquid chromatography–tandem mass spectrometry method(1). A peripheral catheter was placed on the forearm 20 minutes before the test to prevent any influence of the sympathetic system during HUTT. To prevent breakdown of catecholamines, all blood collection tubes were kept cold before and after sampling and samples processed within 30 minutes. Blood sampling was realized at the end of baseline (BP baseline supine continuous recording – green part on the left of each figure depicting HUTT-pv) and after two and five minutes in the upright position. Plasma renin activity and aldosterone were measured to assess the Renin-Angiotensin-Aldosterone-System (RAAS) activity. Basal cortisol level was measured twice to exclude adrenocortical insufficiency.

## Auto-antibody screening

Autoantibodies (AABs) were evaluated using CellTrend GmbH with plasma sampled before administration of IVIG.

# Results

## Day 27 HUTT-pv without stepping and before IVIG (Figure 2, panel A)

The baseline supine vitals were 145/96 mmHg and 86 bpm sinus. During the progressive verticalization, the systolic blood pressure decreased progressively while diastolic blood pressure remained stable. At the end of the third minute, diastolic blood pressure and heart rate dropped sharply reaching 66/52 mmHg and the cardiac output decreased to 40% of baseline. The HUTT-pv was discontinued after five minutes at 70 degrees when the patient showed clinical signs of syncope threat. During the last 15 minutes after returning to supine position, the mean blood pressure was 150/99 mmHg for 78 bpm.

Plasma norepinephrine increased from 1.47 nmol/l at baseline to 3.34 nmol/l at the end of the test whereas plasma epinephrine remained stable. The RAAS was weakly activated with a slight increase in plasma renin activity from 1.0 to 1.3 ng/ml/h with no significant increase in plasma aldosterone.

## Day 42 HUTT-pv without stepping and after IVIG

The systolic blood pressure remained stable during the whole examination except for an initial drop of 15 mmHg in the first 30 seconds, and the diastolic blood pressure increased slightly and progressively from 85 to 95 mmHg. The graph showed a higher variability in the upright position. Heart rate increased from 65 bpm to 97 bpm with an average of 85 bpm when standing, and the cardiac output was decreased by 20 to 25 % on average. Plasma norepinephrine increased from 1.03 to 2.28 nmol/l in 5 minutes (like Day 27-HUTT-pv) whereas epinephrine remained almost identical. Baseline plasma renin activity (0.3 ng/ml/h) and aldosterone (72 pg/ml) were low compared to Day-27 and standing did not stimulate the RAAS.

## Results of HUTT-pv with stepping at Day 27 (Figure 2, panel B) and Day 42

During Day-27 HUTT-pv with stepping, the blood pressure slightly decreased at two minutes in the upright position from 149/99 mmHg to 125/94 mmHg, then steadily increased to 133/98 and 140/102 mmHg at five and ten minutes, respectively. Heart rate increased at two minutes from 82 to 119 bpm (indicating a POTS diagnosis, with a heart rate increase of more than 30 beats/minute within 10 minutes of standing) and decreased slightly to 107 and 96 bpm at five and ten minutes, respectively. Cardiac output did not decrease as much in HUTT-pv without stepping. The patient was able to stay in the vertical position for 15 minutes without syncope but was symptomatic (cerebral Doppler was not performed). The baseline catecholamines were slightly lower than the previous HUTT-pv without stepping. Only norepinephrine increased by 30% from 1.33 to 1.76 nmol/l at five minutes. Plasma renin activity and aldosterone were higher at baseline and increased from 1.6 to 2.2 ng/ml/h and 373 to 415 pg/mL, respectively at five minutes.

During the Day 42 HUTT-pv with stepping, mean systolic and diastolic blood pressure remained the same in the supine and standing positions but with an increased variability. Heart rate increased from 60 bpm at baseline to approximately 80 bpm on average at two minutes. Cardiac output increased by 17% and 31% at two and ten minutes, respectively. Baseline plasma renin activity and aldosterone were low and not stimulated by standing. Plasma epinephrine decreased slightly during verticalization whereas norepinephrine doubled from 1.08 to 2.04 nmol/l at two minutes and decreased to 1.44 at five minutes after verticalization.

## Results of HUTT-pv without stepping three months after discharge (Figure 2, panel C)

The systolic blood pressure remained stable during the whole examination around 125 mmHg while the diastolic slightly increased slightly and progressively from 79 to 95 mmHg. Heart rate also increased from 76 to 94 bpm showing adequate response.

Plasma norepinephrine increased from 1.25 nmol/l at baseline to 1.84 nmol/l at the end of the test whereas plasma epinephrine remained stable. The RAAS was not modified during the test.

## Laboratory Analysis

Laboratory study performed on Day-27 showed electrolytes, kidney function and urine analysis in their normal range. Liver and pancreatic enzymes at the beginning of the stay were slightly increased. Adrenal insufficiency (both corticoadrenal and mineraloadrenal) was excluded according to normal plasma cortisol and aldosterone levels and normal plasma renin activity. Heart failure, myocarditis and thyroid dysfunction were ruled out.

Cerebrospinal fluid analysis was normal. Immunological investigations showed normal IL-2 receptor and conversion enzyme levels. Serology for syphilis, Borrelia burgdorferi, tick-borne encephalitis, hepatitis virus A, B, C, E and HIV were all negative. The QuantiFERON test was negative. Anti-nuclear antibody and antineutrophil cytoplasmic antibodies were in the normal range.

## Extensive assessment

### Thoraco-abdominal CT-scan

There was no evidence of thoracic or abdominal neoplasia.

### Brain MRI

No detectable abnormalities.

### Total medullar MRI

No detectable abnormalities other disc herniations, left C5-C6 without disco-radicular impingement, and left L4-L5 with disco-radicular contact on the left L5-S1 roots. In addition, in L5-S1, posteromedial disc overhang with a wide base and disco-radicular contact on the S1 roots in their lateral S1 roots in their lateral recess.

### Lumbar punctures

|  | ***23.03.2022*** | ***29.03.2022*** | ***Cut-off*** |
| --- | --- | --- | --- |
| *White cells* | 1/µm/L | < 1 | < 5 µm/L |
| *Red cells* | 0/µm/L | < 1 | <5/ µm/L |
| *Proteinorachia* | 414 mg/L | 352 | 150-450 mg/L |
| *Glycorachia* | 2.6 mmol/L | 2.7 | 2.2-3.9 mmol/L |
| *Glycorachia/glycemia ratio* | 0.52 | 0.54 |  |
| *Lactarachia* | 1.2 mmol/L | 1.27 | 1.0-2.9 mmol/L |
| *Protein electrophoresis* | Negative | Negative |  |
| *Bacterial culture* | Negative | Negative |  |

### Blood analysis

|  | ***Patient value*** | ***Cut-off*** |
| --- | --- | --- |
| *Borrelia Burgdorferi*  Index IgG | 0.01 | 0.02 |
| Index IgM | 0.2 | 0.2 – 0.32 |
| *Flavivirus*  Dosage IgG | < 9 | 9.0 – 11.0 |
| Dosage IgM | < 9 | 9.0 – 11.0 |
| *Syphilis* Ig index | < 0.100 | 0.9 – 3.1 |
| *Hepatitis B, A, C, E serology* | All Negative |  |
| *HIV and HTLV serology* | All Negative |  |
| *QuantiFERON Mycobacterium tuberculosis* | Negative |  |
| *SARS-COV2 PCR* | Positive (2,4 e8 copies) |  |
|  |  |  |
| *Protein electrophoresis* | No monoclonal peak detected |  |
| *Rheumatoid factor* | < 11 | < 20 UI/mL |
| *Anti-nuclear factor* | 1/160, spotted | < 1/160 |
| *Anti-nucleoproteins* | Negative |  |
| *Anti-PR3* | < 1 | < 5 UI/mL |
| *Anti-MPO* | < 1 | < 6 UI/mL |
| *CD25 (IL-2R)* | 1161 | 458 – 1997 pg/mL |
| *Anti-Ach Receptor antibodies* | 0.20 | < 0.40 – 0.50 nmol/L |
| *Anti-MOG and Anti-AQP4* | Negative |  |
| *Anti-neuronal antibodies* | Negative |  |
| *Onconeuronal antibodies* | Negative |  |
| *Antiganglioside antibodies* | Negative |  |
|  |  |  |
| *Vitamin B1* | 103.2 | 78 – 143 nmol/L |
| *Vitamin B2* | 220.6 | 174 – 471 nmol/L |
| *Vitamin B6* | 85.8 | 51 – 183 nmol/L |
| *Vitamin B12* | 715 | 145 – 569 pmol/L |
| *Folate* | 24.2 | 8.8 – 60.8 nmol/L |

### Electroencephalogram

We described 11Hz alpha base activity, regular, well-modulated, normo-voiced, well spatialized and bilaterally responsive. In addition, no focusing, no epileptiform element and no clinical manifestation.

### Electroneuromyography (ENMG)

Concerning the symptomatology of the upper right limb, the electro-clinical examination was normal. As for the suspicion of dysautonomia, we were unable to demonstrate this in our complementary examinations. The sympathetic skin reflex and the Sudoscan were normal, showing the integrity of the sympathetic afferents, as was the RR (R wave from the electrocardiogram QRS complex) interval measurement for the parasympathetic afferent. ENMG was performed after IVIG treatment and resolution of the orthostatic hypotension.

## Supplemental figures

### Supplemental figure 1: Day-27 Beat-by-beat measurement of heart rate, blood pressure and cardiac output (Same as figure 2, panel A, but increased zooming in and addition of relative cardiac output to baseline)

**
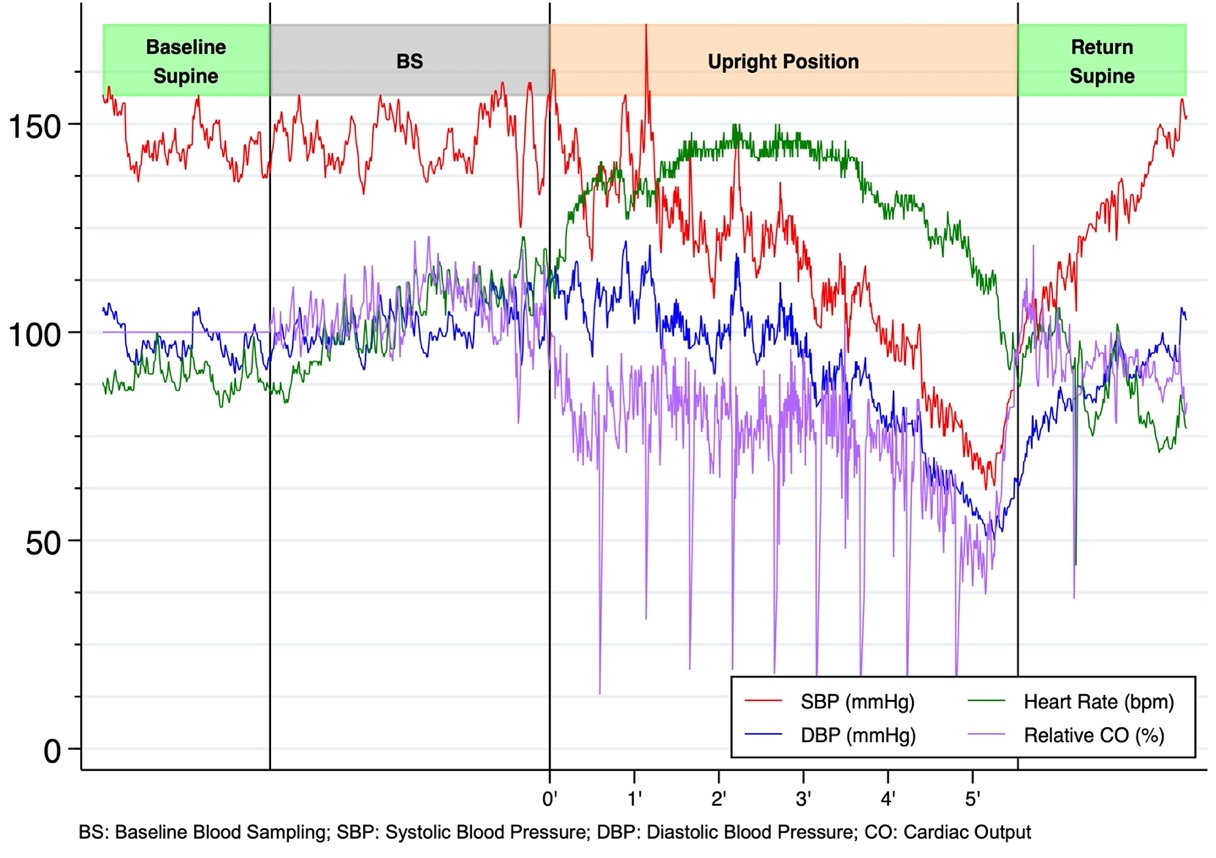
**

# Citations

1. Dunand M, Gubian D, Stauffer M, Abid K, Grouzmann E. High-Throughput and Sensitive Quantitation of Plasma Catecholamines by Ultraperformance Liquid Chromatography–Tandem Mass Spectrometry Using a Solid Phase Microwell Extraction Plate. Anal Chem. 2013 Apr 2;85(7):3539–44.
